# The relationship between rurality, travel time to care and death by suicide

**DOI:** 10.1186/s12888-023-04805-w

**Published:** 2023-05-17

**Authors:** Rebecca Barry, Jürgen Rehm, Claire de Oliveira, Peter Gozdyra, Simon Chen, Paul Kurdyak

**Affiliations:** 1 University of Toronto, University Dr NW, Calgary, AB T2N 1N4 Canada; 2 Centre for Addiction and Mental Health, Moscow, Russian Federation; 3 Dresden University of Technology, Dresden, Germany; 4 ICES, Toronto, Canada; 5 Centre for Health Economics and Hull York Medical School, University of York, York, UK

**Keywords:** Suicide, Healthcare access, Rurality

## Abstract

**Background:**

We previously found an association between rurality and death by suicide, where those living in rural areas were more likely to die by suicide. One potential reason why this relationship exists might be travel time to care. This paper examines the relationship between travel time to both psychiatric and general hospitals and suicide, and then determine whether travel time to care mediates the relationship between rurality and suicide.

**Methods:**

This is a population-based nested case-control study. Data from 2007 to 2017 were obtained from administrative databases held at ICES, which capture all hospital and emergency department visits across Ontario. Suicides were captured using vital statistics. Travel time to care was calculated from the resident’s home to the nearest hospital based on the postal codes of both locations. Rurality was measured using Metropolitan Influence Zones.

**Results:**

For every hour in travel time a male resides from a general hospital, their risk of death by suicide doubles (AOR = 2.08, 95% CI = 1.61–2.69). Longer travel times to psychiatric hospitals also increases risk of suicide among males (AOR = 1.03, 95%CI = 1.02–1.05). Travel time to general hospitals is a significant mediator of the relationship between rurality and suicide among males, accounting for 6.52% of the relationship between rurality and increased risk of suicide. However, we also found that there is effect modification, where the relationship between travel time and suicide is only significant among males living in urban areas.

**Conclusions:**

Overall, these findings suggest that males who must travel longer to hospitals are at a greater risk of suicide compared to those who travel a shorter time. Furthermore, travel time to care is a mediator of the association between rurality and suicide among males.

**Supplementary Information:**

The online version contains supplementary material available at 10.1186/s12888-023-04805-w.

## Background

Travel time to obtain health services has been identified as a potential barrier to health care utilization [1–3]. Travel time to care is considered an important measure of health service accessibility, and is correlated with patient survival rates, where longer travel time to care is associated with lower survival rates [4, 5]. A systematic review on travel time to care indicated that 77% of studies found an association between travel time to care and worse health outcomes including lower survival rates and increased length of stay in hospital [5]. The relationship between travel time and death by suicide has not been examined in depth. Access to health care services may play a role in suicide prevention, as Tondo et al. (2005) found a negative correlation between suicide and the density of psychiatrists and general practitioners in the United States [6]. Previously, we found an association between rurality and suicide among adults living in Ontario, Canada, where those living in rural areas experienced an increased risk of death by suicide [7]. Other studies have also shown this association [8]. While there are several plausible reasons on why this association between rurality and suicide might exist, one possibility is that, because individuals who live in rural regions likely have longer travel times to obtain care, the longer travel time might serve as a barrier and explain part of the relationship between rurality and death by suicide.

This study first aims to examine the relationship between travel time to both psychiatric and general hospitals and death by suicide, and then aims to determine whether travel time to care mediates the relationship between rurality and suicide.

## Methods

### Study design and population

This study uses a nested matched case-control design. Cases are defined as adults (ages 18 and over) living in Ontario who died by suicide between April 1, 2007 and December 31, 2015. Controls are randomly selected age- and sex-matched adults living in Ontario who were alive at the time of the matched control’s death by suicide. Four controls were matched for every suicide case.

### Data sources

Data include administrative databases held at ICES (formerly known as the Institute for Clinical Evaluative Sciences) in Toronto, Ontario. ICES is an independent, non-profit research institute whose legal status under Ontario’s health information privacy law allows it to collect and analyze health care and demographic data, without consent, for health system evaluation and improvement. The databases used for this analysis include the Registered Persons Database (RPDB), which includes data on individual’s age, sex and eligibility for public health care insurance and used to capture cases and controls; the Canadian Institute for Health Information National Ambulatory Care Reporting System (CIHI-NACRS), which includes information on emergency department (ED) visits, including prior suicide attempts; the 2016 Postal Code Conversion File (PCCF) for geocoding of hospital postal codes; 2018 road data accessed as open data through Land Information Ontario portal to calculate drive times; Census data, which provide information on neighbourhood-level income expressed as quintiles and the Ontario Marginalization Index (ON-MARG), which includes data on area-level residential instability and dependency; the Immigration, Refugees and Citizenship Canada (IRCC)’s Permanent Resident Database, which provides information on migrant status; and Vital Statistics Death data from the Office of the Registrar General (ORG-D), which provide information on date and cause of death, including suicide-related deaths. The Vital Statistics cause of death field has been found to have over 95% sensitivity when compared to coroner-confirmed suicides, [9] and the algorithm for suicide attempts has also been validated [10]. These datasets were linked using unique encoded identifiers and analyzed at ICES, in accordance with Ontario privacy legislation.

### Outcome

The outcome of interest is death by suicide, which is ascertained using International Classification of Diseases (ICD), Ninth and Tenth Revision (ICD-9 and ICD-10), codes for suicide (ICD-9 E950-E959; ICD-10 × 60-X84) or possible suicide (ICD-10 Y10-Y19, Y28) and validated using coroner’s data [9]. This definition is based on the standardization document for ICES at the time of study commencement, taking into account the sensitivity and specificity of these codes in the Ontario population.

### Aim 1 exposure: travel time to care

Travel time to general and psychiatric hospitals are the primary exposures. Drive travel time is calculated from the resident’s home to the nearest hospital based on the postal codes of both locations. This analysis uses ArcGIS v. 10.2 by ESRI [11]. No traffic congestion or other travel impedances were assumed. This measure considers road networks, rather than measuring direct point-to-point distance measures from the individual’s postal code to the hospital. There are 5 psychiatric hospitals in Ontario, and 171 general hospitals. Travel time is measured in minutes and then also converted into hours.

### Aim 2 exposure: rurality

The exposure is rurality, which is measured using Metropolitan Influence Zones (MIZ) [12]. MIZs are a measure of rurality that considers population size and access to urban centre labour markets. A MIZ where the population is larger than 10,000 people is considered urban [12]. Levels of rurality are determined by those commuting to work in a census metropolitan agglomeration (CMA). A metropolitan influence zone (MIZ) is considered strong when 30% or more individuals work in the CMA, moderate (5–30%), weak (1–5%), and remote ( = < 40 people) [12].

### Aim 2 mediation variable: travel time to care

For the second aim, travel time to care is the mediator. It is defined based on road networks between the individual’s residence and the nearest hospital, as described above.

### Covariates

The Johns Hopkins Adjusted Clinical Groups (ACGs) ® system version 10 is used to categorize chronic conditions into 32 Aggregate Diagnosis Groups (ADGs) [13]. These 32 disease classifications have been validated for use in predicting mortality among a population-based cohort of adults with schizophrenia in Ontario, Canada [13]. Examples of ADGs include dermatologic conditions, chronic stable and unstable conditions, and time-limited conditions. We also included migrant status, the Ontario Marginalization Indices of instability and dependency, [14] neighbourhood income quintile, and any prior suicide attempt. Prior suicide attempts were defined based on emergency department presentation with self-harm codes X60-X84, Y10-Y19, Y28 [10].

### Statistical analyses

We first compared patient characteristics by cases and controls using standardized differences, because we have a large sample size and standardized differences measures the effect size independent of sample size [15]. We then calculated the median travel times and interquartile ranges (IQRs) to general hospitals and psychiatric hospitals among cases and controls of both sexes, and compared them using the Wilcoxon test. We then used multiple logistic regression to model the relationship between travel time to hospitals and death by suicide. In addition, we used multiple logistic regression to analyze the relationship between travel time to psychiatric hospitals and death by suicide. All covariates are included in the model to allow for comparability between effect estimates.

**Fig. 1:**
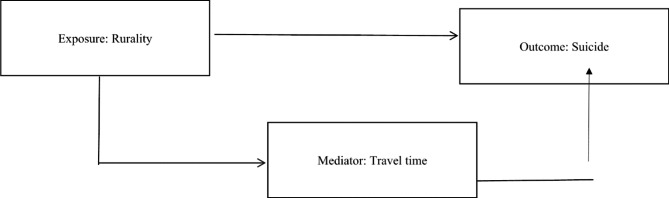
Mediation Analysis

For the mediation analysis, the exposure is rurality, the outcome is death by suicide, and the mediating variable is travel time to general or psychiatric hospital (Fig. 1). To examine travel time as a mediator of the relationship between rurality and suicide, we used multiple logistic regression and the CAUSALMED procedure in SAS to derive the natural direct effect (NDI), the natural indirect effect (NDE), and the percent mediation [16]. This procedure is based on VanderWeele’s mediation approach based on the counterfactual framework. The natural direct effect is the conditional association between the exposure and the outcome, whereas the natural indirect effect is the combination of exposure’s effect on the mediator, and the mediator’s effect on the outcome [17, 18]. We used the case control option in SAS to fit the mediator model by only using observations for those in the control group [16]. We generated a 95% bootstrap Wald confidence interval for the effect estimates, using 1,000 bootstrap samples with a seed value of 740,404 (based on a random number generator).

Finally, we also examined the possibility of effect modification, by including the interaction term between rurality and travel time in the model.

All analyses were completed using SAS Enterprise version 7.1.

### Role of the funding source

The analyses, conclusions, opinions, and statements expressed herein are solely those of the authors and do not reflect those of the funding or data sources.

## Results

Table 1 shows a comparison of cases (N = 9,848) and controls (N = = 39,392) by patient characteristics. Cases were more likely to be non-immigrants, more likely to live in lower income neighbourhoods, and more likely to live in neighbourhoods with higher instability indices. Furthermore, cases were more likely to have most ADG diagnoses. Finally, cases were more likely than controls to have a prior suicide attempt.

**Table 1:** Study Population Characteristics

Variables	Cases (N = 9,848) N (%)	Controls (N = 39,392) N (%)	Standardized Difference
Age (mean, SD)	48.73 (16.42)	48.74 (16.42)	-0.001
Sex (% female)	2608 (26.48)	10,432 (26.48)	0
Migration status			
Non-immigrant	9017 (91.56)	32,827 (83.33)	0.250
Immigrant	622 (6.32)	5412 (13.74)	0.249
Refugee	209 (2.12)	1153 (2.93)	0.051
Neighbourhood Income Quintile			
Q1 (lowest)	2523 (25.83)	7222 (18.41)	0.177
Q2	2092 (21.42)	7716 (19.67)	0.041
Q3	1898 (19.43)	7898 (20.14)	0.020
Q4	1688 (17.28)	8218 (20.95)	0.095
Q5 (highest)	1565 (16.02)	8169 (20.83)	0.126
Neighbourhood Instability Index Quintile			
1 (lowest instability)	1293 (13.37)	8236 (21.08)	0.208
2	1542 (15.95)	7628 (19.52)	0.098
3	1769 (18.30)	7303 (18.69)	0.015
4	2007 (20.76)	7416 (18.98)	0.039
5 (highest instability)	3057 (31.62)	8489 (21.73)	0.217
Dependency Quintile			
1 (lowest dependency)	2082 (21.53)	9389 (24.03)	0.065
2	1833 (18.96)	7892 (20.20)	0.036
3	1771 (18.32)	7436 (19.03)	0.023
4	1865 (19.29)	6922 (17.72)	0.035
5 (highest dependency)	2117 (21.90)	7433 (19.02)	0.066
Time Limited Minor (ADG1)	2528 (25.67)	8040 (20.41)	0.125
Time Limited Minor: Primary Infection (ADG2)	4242 (43.07)	15,294 (38.83)	0.087
Time Limited Major (ADG3)	1476 (14.99)	2063 (5.24)	0.328
Time Limited Major: Primary Infection (ADG4)	1697 (17.23)	3346 (8.49)	0.263
Allergies (ADG5)	543 (5.51)	2242 (5.69)	-0.007
Asthma (ADG6)	620 (6.30)	1737 (4.41)	0.084
Likely to recur: Discrete (ADG7)	3808 (38.67)	11,361 (28.84)	0.209
Likely to recur: Discrete infection (ADG8)	1891 (19.20)	5709 (14.49)	0.126
Likely to recur Progressive (ADG9)	852 (8.65)	1017 (2.58)	0.266
Chronic Medical: Stable (ADG10)	4635 (47.07)	15,865 (40.27)	0.137
Chronic Medical: Unstable (ADG11)	3194 (32.43)	7254 (18.41)	0.326
Chronic Specialty: Stable-Orthopedic (ADG12)	338 (3.43)	991 (2.52)	0.054
Chronic Specialty: Stable-Ear, Nose, Throat (ADG13)	267 (2.71)	833 (2.11)	0.039
Chronic Specialty-Stable-Eye (ADG14)	560 (5.69)	1958 (4.97)	0.032
Chronic Specialty: Unstable-Orthopedic (ADG16)	418 (4.24)	874 (2.22)	0.115
Chronic Specialty: Unstable-Ear, Nose, Throat (ADG17)	< 10	< 10	-0.01
Chronic Specialty: Unstable-Eye (ADG18)	579 (5.88)	2063 (5.24)	0.028
Dermatologic (ADG20)	1170 (11.88)	4981 (12.64)	-0.023
Injuries/Adverse effects: Minor (ADG21)	3277 (30.60)	7431 (18.86)	0.333
Injuries/Adverse effects: Major (ADG22)	3904 (39.64)	5665 (14.38)	0.593
Psychosocial: Time Limited, Minor (ADG23)	1476 (14.99)	1431 (3.63)	0.398
Psychosocial: Recurrent or Persistent, Stable (ADG24)	6338 (64.36)	8440 (21.43)	0.963
Psychosocial: Recurrent or Persistent, Unstable (ADG25)	3755 (38.13)	2094 (5.32)	0.867
Signs/Symptoms: Minor (ADG26)	4464 (45.33)	12,071 (30.64)	0.306
Signs/Symptoms: Uncertain (ADG27)	5932 (60.24)	17,291 (43.89)	0.332
Signs/Symptoms: Major (ADG28)	3750 (38.08)	9456 (24.00)	0.308
Discretionary (ADG29)	1930 (19.60)	6282 (15.95)	0.096
See and Reassure (ADG30)	243 (2.47)	802 (2.04)	0.029
Prevention/Administrative (ADG31)	3327 (33.78)	13,150 (33.38)	0.008
Malignancy (ADG32)	928 (9.42)	2673 (6.79)	0.097
Pregnancy (ADG33)	123 (1.25)	625 (1.59)	0.029
Dental (ADG34)	386 (3.92)	654 (1.66)	0.138
Prior suicide attempt	1832 (18.60)	218 (0.55)	0.644

**Fig. 2:**
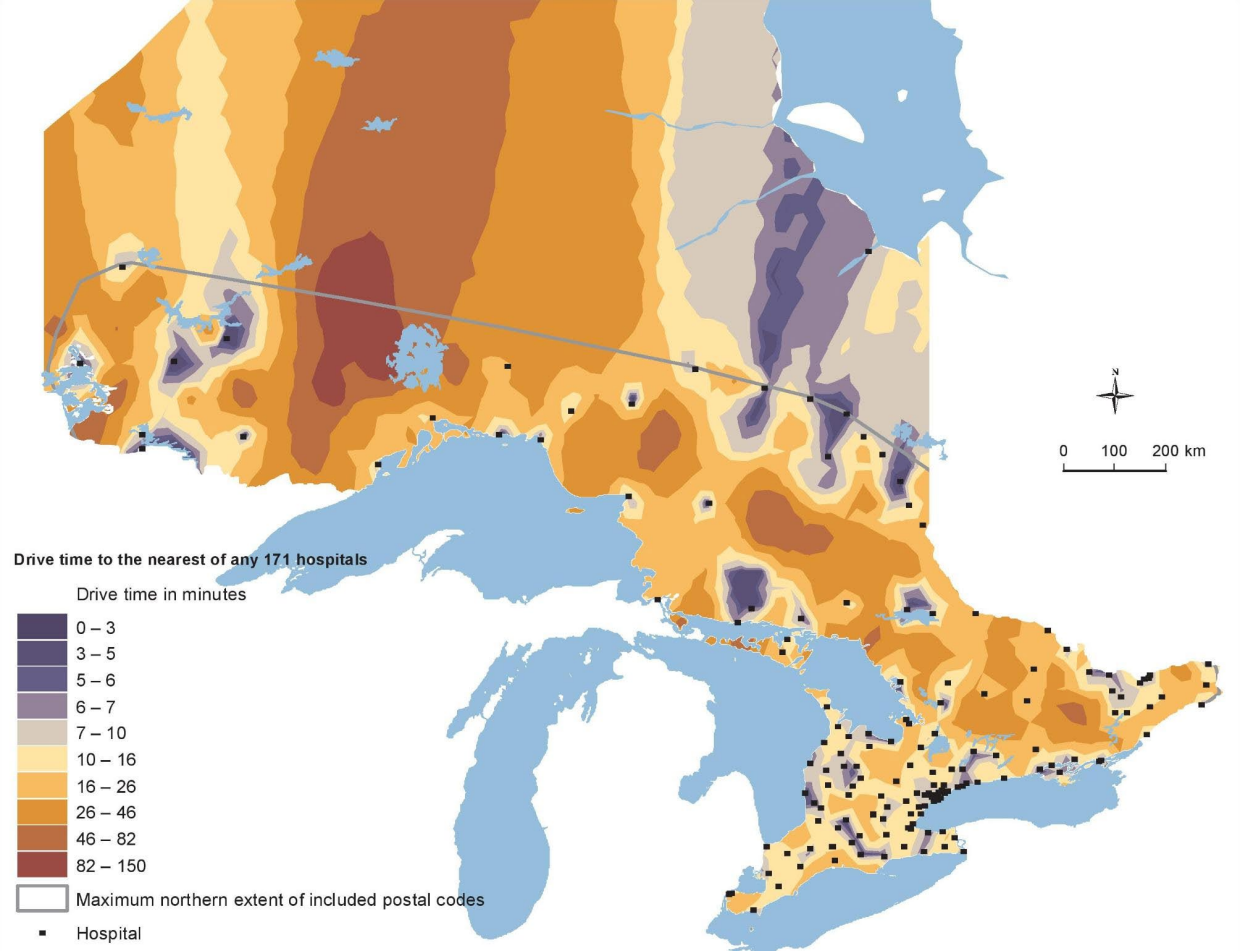
GIS analysis: Travel time to General Hospitals

**Fig. 3:**
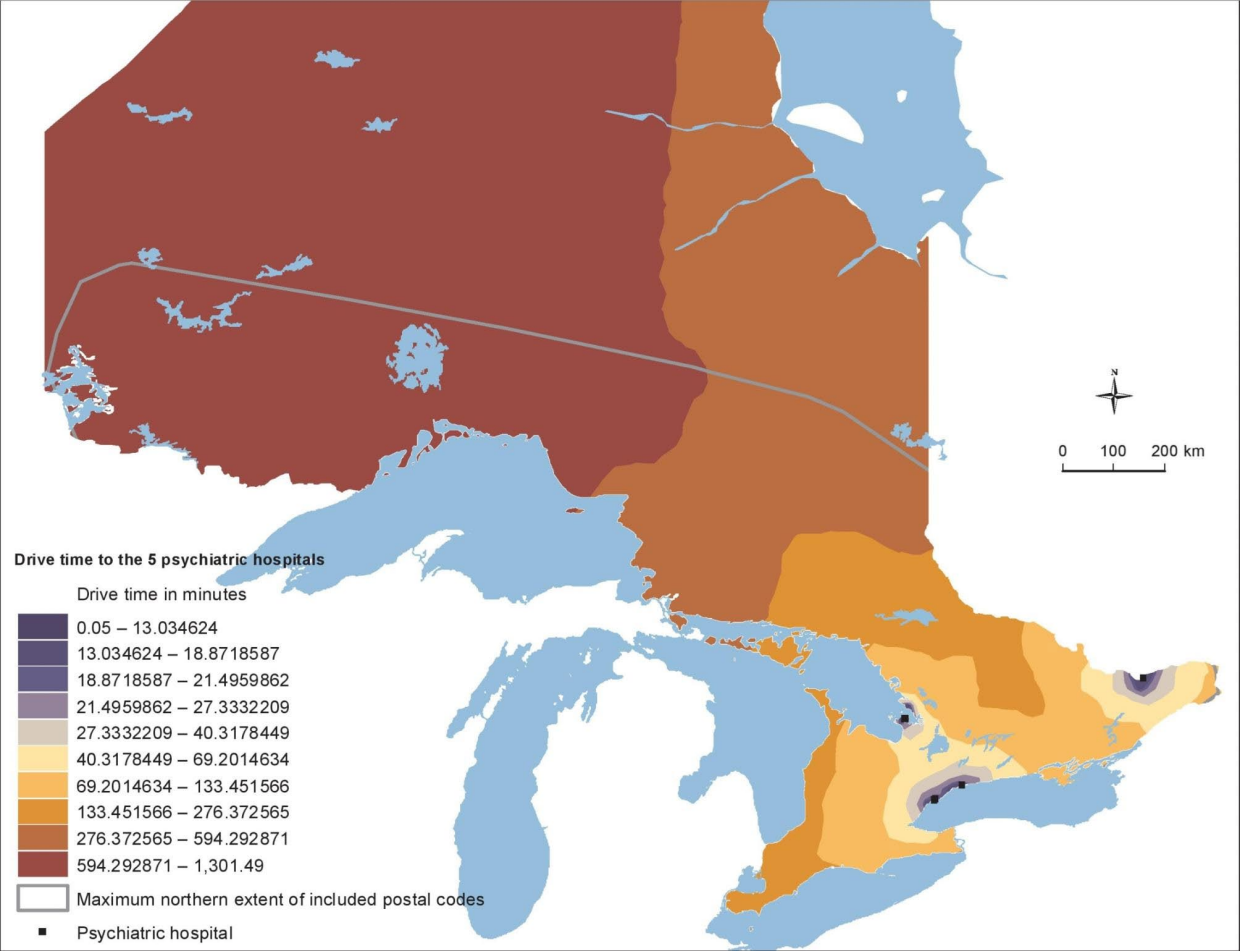
GIS Analysis: Travel time to Psychiatric Hospitals

Generally, travel time ranges from 0 min to 2.5 h for general hospitals (Fig. 2), and from 0 min to over 16 h for psychiatric hospitals (Fig. 3). Supplementary Table 1 indicates that males who died by suicide had to travel significantly longer to psychiatric hospitals (50.98 min vs. 36.40 min) compared to controls. However, cases of both sexes traveled shorter times to general hospitals compared to controls, although all groups have a median travel time ranging from 6.3 to 7.4 min. There were no significant differences in travel times for psychiatric hospitals between female cases and controls.

Table 2 indicates that males who need to travel longer times to hospitals have double the risk of suicide for every hour they live further away from a general hospital (AOR = 2.08, 95% CI (confidence interval) = 1.61–2.69). Furthermore, males living further in travel time from psychiatric hospitals were also more likely to die by suicide (AOR = 1.03, 95% CI = 1.02–1.05). The association between travel time and suicide is not statistically significant among females.

**Table 2:** Multiple logistic regression predicting suicide [1]

	Hours OR (95% CI)
*General Hospital*	
Males: Death by suicide	2.08 (1.61, 2.69)
Females: Death by suicide	1.29 (0.71–2.36)
*Psychiatric Hospital*	
Males: Death by suicide	1.03 (1.02, 1.05)
Females: Death by suicide	1.00 (0.97, 1.03)

1. Adjusted for neighbourhood income quintile, migration status, dependency and instability marginalization indices measured at the neighbourhood level and Johns Hopkins ADGs, and prior suicide attempt

Legend: OR – odds ratio; CI – confidence interval; ADGs – Aggregate Diagnosis Groups

Travel times to general hospitals were greatest among rural areas with strong metropolitan influences (Supplementary Table 2). Urban populations tend to have lower travel times to hospitals while the most rural areas with the least metropolitan influence also had lower travel times. Travel times to psychiatric hospitals increased with increasing rurality (Supplementary Table 3).

Table 3 indicates that travel time to general hospitals is a significant mediator of the relationship between rurality and suicide among males and mediates 6.52% of this association. This means that 6.52% of the total relationship between rurality and suicide among males is accounted for by travel time to general hospitals. Travel time to general hospitals is not a significant mediator among females. Travel time to psychiatric hospitals is not a significant mediator of the relationship between rurality and suicide among either sex.

**Table 3:** Mediation Analysis

	NDE (95% CI)	NIE (95% CI)	% mediated (95% CI)
*General Hospital*			
Males: Death by suicide	0.015 (0.012, 0.017)	0.001 (0.00002, 0.002)	6.52 (0.11, 12.94)
Females: Death by suicide	0.002 (-0.003, 0.006)	0.0005 (-0.001, 0.002)	22.08 (-225.43, 362.71)
*Mental Health Hospital*			
Males: Death by suicide	0.015 (0.012, 0.018)	0.001 (-0.0002, 0.002)	6.35 (-1.46, 14.19)
Females: Death by suicide	0.003 (-0.018, 0.0071)	-0.0007 (-0.003, 0.001)	-31.57 (-351.96, 560.53)

Legend: NDE – Natural direct effect; NIE – Natural indirect effect; CI – confidence intervals

Supplementary Table 8 indicates that there is effect modification, where the relationship between travel time to care and suicide is significant among males living in urban areas but not among males living in rural areas.

## Interpretation

Our findings indicate that males are at an increased risk of suicide when they have longer travel times to obtain care. For every 60 min in travel time a male resides from a general hospital, their risk of death by suicide doubles (AOR = 2.08, 95% CI = 1.61–2.69). To a much lesser degree, longer travel times to psychiatric hospitals also increases risk of suicide among males (AOR = 1.03, 95%CI = 1.02–1.05). Our findings also suggest that travel time to general hospitals is a significant mediator of the relationship between rurality and suicide among males, accounting for 6.52% of the relationship between rurality and increased risk of suicide. Finally, there is some effect modification where the relationship between travel time to care and suicide is significant only among males living in urban areas. Overall, these findings suggest there is an important sex difference, where males that must travel longer to care are at a greater risk of suicide compared to those who travel shorter times.

Our findings differ from an American study that examined the relationship between the distance to Veteran Affairs psychiatric hospitals and death by suicide among veterans. They found that distance was not a predictor of death by suicide [19]. However, this study used a more specific veteran study population. Moreover, it also uses point-to-point straight-line distances rather than travel time considering road networks and speed limits. These differences may explain the differences in study findings.

Other studies have examined the relationship between travel time to care and survival for particular diseases (e.g., cancer, kidney disease); 77% of these studies found an association between increased travel distance and adverse health outcomes [5]. Only one study examined travel time to care and mental health-related outcomes; this study found that as distance to care increased, the seriousness of a mental health-related diagnosis increased [20].

Two studies have examined the relationship between travel distance to emergency departments and suicide attempts and found that travel time was negatively associated with risk of suicide attempts.^[[21]–[22]]^ However, this may be due to a greater likelihood of presenting at an emergency department for less severe injuries if an individual lives closer to the emergency department, as one subgroup analysis indicates that the relationship is strongest for self-harm involving minor self-cutting [21].

As there are few studies that have examined the relationship between travel time to care and suicide, it is uncertain why the association exists among males and not females. However, previous studies have shown that the relationship between rurality and suicide is only significant among males, and suggested reasons include: cultural norms and attitudes towards masculinity in a rural setting, occupational risks that may be higher among males in rural settings, or that emotionally supportive relationships are more protective against major depression for women than for men [7].

This study has strengths. First, the network analysis considers road networks rather than calculating distance based on straight-line distances from residential postal codes to hospitals. Travel times are a more appropriate measure of transportation barriers than population density or straight-line distances, and it has been shown that these measures can provide different results when used as predictor variables [4, 23]. Second, this study includes all recorded suicide cases in Ontario over an eight-year period. This large sample size provides this study with the required power to detect smaller differences between cases and controls. Third, this study is population-based and the data capture health care service use for virtually all people living in the province of Ontario as individuals cannot opt out, thus decreasing the potential for selection bias and loss-to-follow-up. Fourth, there is a high degree of variability in travel time to care in Ontario, Canada. This allows for a high degree of variability in the exposure of interest. Fifth, data quality is considered to be high because data are routinely collected and undergo quality assessment by analysts.

One limitation of this analysis is that travel times do not consider congestion and are based on optimal driving conditions. The analysis does not account for transit routes that may be available in urban areas. However, most people living in Ontario (78%) use private vehicles to go to work [24]. A second limitation is the potential for misclassification based on cause of death. For example, a suicide may be recorded as accidental and not included in the study. However, this is not expected to differ by travel time to hospitals. A third limitation is that survival rates following a suicide attempt may be lower among those living further from a hospital as they may be more likely to succumb to their injuries during transit. One study found that in cases of emergency, a 10 km increase in distance is associated with a 1% increase in mortality [25]. However, if this was driving the association between travel time and suicide, we would expect to see a similar association among females. It is also unlikely that this relatively smaller increase in mortality would account for the large differences in suicides among males. A fourth limitation is that we do not have access to Indigenous status or sexual orientation, which are potential effect modifiers of the association. That is, the magnitude of the effect of travel time on risk of suicide may differ in these populations. A fifth limitations are that individuals with no road access to care are excluded from the analysis because travel time to care cannot be determined given the assumptions and GIS approach used for this analysis. Finally, there may be additional factors that increase risk of suicide as well as the likelihood of someone choosing to reside in a remote area that may not be captured in our data. Therefore, there is a possibility of unmeasured confounding.

While it is not feasible to decrease travel times to hospitals in many cases, this increase in risk is concerning and should be addressed in other ways. For example, travelling clinics that visit areas with no local hospitals should aim to target males, with particular attention paid to mental health concerns. In addition, there could be a focus on supporting patients with referrals to continuing supports (i.e., telepsychiatry, local counselling, etc.) and follow-up to ensure patients have made use of the referrals or to give alternative options where needs are unmet. In areas that are far from hospitals, educating rural physicians on how to identify suicidal ideation and how to refer patients to effective long-term mental health care is also needed. It appears that those living furthest from general hospitals are individuals who live in rural areas within commuting distance of metropolitan areas. While supports should be aimed at people living in the most rural areas as they are at greater risk of suicide than those living in urban areas, particular attention should also be directed at those living in rural areas close to metropolitan areas with regards to travel time to hospitals. However, the finding that rurality may be an effect modifier of the relationship between travel time and suicide suggests that there should be a particular focus on individuals living in urban areas but with the longest travel times to care.

Overall, this study demonstrates that the risk of death by suicide among males increases with increasing travel time to hospitals and, to a lesser extent, to psychiatric hospitals, and that the travel time to hospitals is a significant mediator and effect modifier of the relationship between rurality and increased risk of suicide.

## Electronic supplementary material

Below is the link to the electronic supplementary material.

Supplementary Material 1

## Data Availability

The deidentified dataset from this study is held securely in coded form at ICES. While legal data sharing agreements between ICES and data providers (e.g., healthcare organizations and government) prohibit ICES from making the dataset publicly available, access may be granted to those who meet pre-specified criteria for confidential access, available at www.ices.on.ca/DAS (email: das@ices.on.ca). The full dataset creation plan and underlying analytic code are available from the authors upon request, understanding that the computer programs may rely upon coding templates or macros that are unique to ICES and are therefore either inaccessible or may require modification.
