# Supplementary material for: The relationship between rurality, travel time to care and death by suicide
Source: BMC Psychiatry. 2023 May 17;23:345. doi: 10.1186/s12888-023-04805-w (PMC10189916; doi:10.1186/s12888-023-04805-w)
Supplement: Supplementary file 1 — Supplementary Material 1 [file 12888_2023_4805_MOESM1_ESM.docx]

Supplementary Table 1: Study Population Characteristics

| Variables | Cases  (N=9,848) | Controls (N=39,392) | Standardized Difference |
| --- | --- | --- | --- |
| Age | 48.73 (16.42) | 48.74 (16.42) | -0.001 |
| Sex (% female) | 2608 (26.48) | 10432 (26.48) | 0 |
| Migration status |  |  |  |
| Non-immigrant | 9017 (91.56) | 32827 (83.33) | 0.250 |
| Immigrant | 622 (6.32) | 5412 (13.74) | 0.249 |
| Refugee | 209 (2.12) | 1153 (2.93) | 0.051 |
| Neighbourhood Income Quintile |  |  |  |
| Q1 (lowest) | 2523 (25.83) | 7222 (18.41) | 0.177 |
| Q2 | 2092 (21.42) | 7716 (19.67) | 0.041 |
| Q3 | 1898 (19.43) | 7898 (20.14) | 0.020 |
| Q4 | 1688 (17.28) | 8218 (20.95) | 0.095 |
| Q5 (highest) | 1565 (16.02) | 8169 (20.83) | 0.126 |
| Neighbourhood Instability Index Quintile |  |  |  |
| 1 (lowest instability) | 1293 (13.37) | 8236 (21.08) | 0.208 |
| 2 | 1542 (15.95) | 7628 (19.52) | 0.098 |
| 3 | 1769 (18.30) | 7303 (18.69) | 0.015 |
| 4 | 2007 (20.76) | 7416 (18.98) | 0.039 |
| 5 (highest instability) | 3057 (31.62) | 8489 (21.73) | 0.217 |
| Dependency Quintile |  |  |  |
| 1 (lowest dependency) | 2082 (21.53) | 9389 (24.03) | 0.065 |
| 2 | 1833 (18.96) | 7892 (20.20) | 0.036 |
| 3 | 1771 (18.32) | 7436 (19.03) | 0.023 |
| 4 | 1865 (19.29) | 6922 (17.72) | 0.035 |
| 5 (highest dependency) | 2117 (21.90) | 7433 (19.02) | 0.066 |
| Time Limited Minor (ADG1) | 2528 (25.67) | 8040 (20.41) | 0.125 |
| Time Limited Minor: Primary Infection (ADG2) | 4242 (43.07) | 15294 (38.83) | 0.087 |
| Time Limited Major (ADG3) | 1476 (14.99) | 2063 (5.24) | 0.328 |
| Time Limited Major: Primary Infection (ADG4) | 1697 (17.23) | 3346 (8.49) | 0.263 |
| Allergies (ADG5) | 543 (5.51) | 2242 (5.69) | -0.007 |
| Asthma (ADG6) | 620 (6.30) | 1737 (4.41) | 0.084 |
| Likely to recur: Discrete (ADG7) | 3808 (38.67) | 11361 (28.84) | 0.209 |
| Likely to recur: Discrete infection (ADG8) | 1891 (19.20) | 5709 (14.49) | 0.126 |
| Likely to recur Progressive (ADG9) | 852 (8.65) | 1017 (2.58) | 0.266 |
| Chronic Medical: Stable (ADG10) | 4635 (47.07) | 15865 (40.27) | 0.137 |
| Chronic Medical: Unstable (ADG11) | 3194 (32.43) | 7254 (18.41) | 0.326 |
| Chronic Specialty: Stable-Orthopedic (ADG12) | 338 (3.43) | 991 (2.52) | 0.054 |
| Chronic Specialty: Stable-Ear, Nose, Throat (ADG13) | 267 (2.71) | 833 (2.11) | 0.039 |
| Chronic Specialty-Stable-Eye (ADG14) | 560 (5.69) | 1958 (4.97) | 0.032 |
| Chronic Specialty: Unstable-Orthopedic (ADG16) | 418 (4.24) | 874 (2.22) | 0.115 |
| Chronic Specialty: Unstable-Ear, Nose, Throat (ADG17) | <10 | <10 | -0.01 |
| Chronic Specialty: Unstable-Eye (ADG18) | 579 (5.88) | 2063 (5.24) | 0.028 |
| Dermatologic (ADG20) | 1170 (11.88) | 4981 (12.64) | -0.023 |
| Injuries/Adverse effects: Minor (ADG21) | 3277 (30.60) | 7431 (18.86) | 0.333 |
| Injuries/Adverse effects: Major (ADG22) | 3904 (39.64) | 5665 (14.38) | 0.593 |
| Psychosocial: Time Limited, Minor (ADG23) | 1476 (14.99) | 1431 (3.63) | 0.398 |
| Psychosocial: Recurrent or Persistent, Stable (ADG24) | 6338 (64.36) | 8440 (21.43) | 0.963 |
| Psychosocial: Recurrent or Persistent, Unstable (ADG25) | 3755 (38.13) | 2094 (5.32) | 0.867 |
| Signs/Symptoms: Minor (ADG26) | 4464 (45.33) | 12071 (30.64) | 0.306 |
| Signs/Symptoms: Uncertain (ADG27) | 5932 (60.24) | 17291 (43.89) | 0.332 |
| Signs/Symptoms: Major (ADG28) | 3750 (38.08) | 9456 (24.00) | 0.308 |
| Discretionary (ADG29) | 1930 (19.60) | 6282 (15.95) | 0.096 |
| See and Reassure (ADG30) | 243 (2.47) | 802 (2.04) | 0.029 |
| Prevention/Administrative (ADG31) | 3327 (33.78) | 13150 (33.38) | 0.008 |
| Malignancy (ADG32) | 928 (9.42) | 2673 (6.79) | 0.097 |
| Pregnancy (ADG33) | 123 (1.25) | 625 (1.59) | 0.029 |
| Dental (ADG34) | 386 (3.92) | 654 (1.66) | 0.138 |
| Prior suicide attempt | 1832 (18.60) | 218 (0.55) | 0.644 |

Supplementary Table 2: Median Travel times to General Hospitals and Psychiatric Hospitals

|  | **Females** |  |  | **Males** |  |  |
| --- | --- | --- | --- | --- | --- | --- |
|  | **Cases** | **Controls** |  | **Cases** | **Controls** |  |
|  | **Median (IQR)** | **Median (IQR)** | **Wilcoxon Test** | **Median (IQR)** | **Median (IQR)** | **Wilcoxon Test** |
| Median Travel Time to General Hospital (minutes) | 6.34  (3.84, 9.84) | 7.33  (4.56, 11.18) | <0.001 | 7.05  (4.17, 11.49) | 7.37  (4.65, 11.21) | 0.0001 |
| Median Travel Time to Psychiatric Hospital (minutes) | 45.05  (18.91, 87.67) | 37.37  (20.50, 82.71) | 0.736 | 50.98  (2.08, 102.80) | 36.40  (19.96, 82.19) | <0.001 |

Legend: IQR – interquartile range

**Supplementary Table 3: Median Travel Times to General Hospitals**

|  | **Females** |  |  |  | **Males** |  |  |  |
| --- | --- | --- | --- | --- | --- | --- | --- | --- |
|  | **Cases** |  | **Controls** |  | **Cases** |  | **Controls** |  |
| **MIZ** | **Median** | **IQR** | **Median** | **IQR** | **Median** | **IQR** | **Median** | **IQR** |
| Urban: POP 1, 500, 000+ | 5.89 | 3.96-8.40 | 6.35 | 4.31-8.77 | 6.10 | 4.05-8.55 | 6.35 | 4.37-8.81 |
| Urban: POP 500, 000-1,499, 999 | 6.70 | 3.97-9.34 | 9.00 | 5.18-12.16 | 7.08 | 4.09-10.40 | 8.53 | 5.11-11.55 |
| Urban: POP 100, 000-499, 999 | 6.88 | 3.97-10.56 | 8.26 | 5.01-11.52 | 7.59 | 4.52-11.26 | 8.59 | 5.25-12.05 |
| Urban: POP 10, 000-99, 999 | 4.45 | 2.94-7.25 | 6.13 | 3.57-11.46 | 5.04 | 2.99-11.20 | 5.88 | 3.51-11.68 |
| Rural: Strong MIZ | 17.37 | 10.40-2.19 | 17.00 | 11.84-22.00 | 16.81 | 11.04-23.04 | 16.28 | 11.55-22.86 |
| Rural: Moderate MIZ | 15.19 | 5.57-25.12 | 15.24 | 8.22-22.70 | 15.86 | 8.39-22.62 | 15.74 | 7.57-24.52 |
| Rural: Weak MIZ | 2.98 | 2.03-4.63 | 6.63 | 2.47-16.68 | 4.84 | 22.09-16.60 | 4.72 | 2.38-16.51 |

Legend: MIZ – Metropolitan Influence Zones; IQR – Interquartile range; POP – population size

Supplementary Table 4: Median Travel times to Psychiatric Hospitals

|  | **Females** |  |  |  | **Males** |  |  |  |
| --- | --- | --- | --- | --- | --- | --- | --- | --- |
|  | **Cases** |  | **Controls** |  | **Cases** |  | **Controls** |  |
| **MIZ** | **Median** | **IQR** | **Median** | **IQR** | **Median** | **IQR** | **Median** | **IQR** |
| Urban: POP 1, 500, 000+ | 20.69 | 13.40-27.72 | 29.04 | 16.18-29.04 | 20.79 | 13.42-28.39 | 22.49 | 15.71-29.04 |
| Urban: POP 500, 000-1,499, 999 | 35.56 | 11.39-46.57 | 26.07 | 14.25-47.08 | 36.46 | 13.16-46.90 | 24.42 | 13.69-46.76 |
| Urban: POP 100, 000-499, 999 | 81.78 | 64.53-130.39 | 80.86 | 63.98-132.12 | 81.9 | 65.03-165.56 | 81.03 | 64.20-136.99 |
| Urban: POP 10, 000-99, 999 | 97.93 | 71.68-181.56 | 100.25 | 75.99-187.03 | 98.23 | 70.59-185.24 | 99.43 | 76.66-184.43 |
| Rural: Strong MIZ | 80.73 | 57.74-124.11 | 76.77 | 54.21-108.80 | 76.73 | 54.21-110.20 | 73.83 | 51.94-99.94 |
| Rural: Moderate MIZ | 115.5 | 78.62-148.94 | 112 | 75.53-143.76 | 111.1 | 77.75-137.80 | 114.25 | 82.23-148.73 |
| Rural: Weak MIZ | 163.76 | 132.66-597.36 | 157.78 | 132.05-679.17 | 363.95 | 133.70-609.80 | 168.15 | 132.66-679.17 |

Legend: MIZ – Metropolitan Influence Zones; IQR – Interquartile range; POP – population si

**Supplementary Table 5: Descriptive Statistics (Male only)**

| Variables | Cases  (N=7,240) | Controls (N=28,960) | Standardized Difference |
| --- | --- | --- | --- |
| Age | 48.87 (16.55) | 48.87 (16.55) | 0 |
| Migration status |  |  |  |
| Non-immigrant | 6660 (92.00) | 24147 (83.38) | 0.264 |
| Immigrant | 421 (5.810 | 3919 (13.53) | 0.263 |
| Refugee | 159 (2.20) | 894 (3.09) | 0.056 |
| Neighbourhood Income Quintile |  |  |  |
| Q1 (lowest) | 1769 (24.64) | 5307 (18.40) | 0.149 |
| Q2 | 1551 (21.61) | 5701 (19.77) | 0.043 |
| Q3 | 1439 (20.05) | 5783 (20.05) | 0.002 |
| Q4 | 1262 (17.58) | 6052 (20.98) | 0.088 |
| Q5 (highest) | 1157 (16.12) | 5997 (20.79) | 0.122 |
| Neighbourhood Instability Index Quintile |  |  |  |
| 1 (lowest instability) | 991 (13.93) | 6033 (21.00) | 0.190 |
| 2 | 1161 (16.32) | 5659 (19.70) | 0.092 |
| 3 | 1353 (19.01) | 5359 (18.66) | 0.005 |
| 4 | 1502 (21.11) | 5427 (18.89) | 0.050 |
| 5 (highest instability) | 2109 (29.64) | 6246 (21.74) | 0.175 |
| Dependency Quintile |  |  |  |
| 1 (lowest dependency) | 1460 (20.52) | 6909 (24.05) | 0.089 |
| 2 | 1356 (19.14) | 5793 (20.17) | 0.030 |
| 3 | 1322 (18.58) | 5542 (19.29) | 0.023 |
| 4 | 1438 (20.21) | 5110 (17.79) | 0.057 |
| 5 (highest dependency) | 1534 (21.56) | 5370 (18.70) | 0.066 |
| Time Limited Minor (ADG1) | 1616 (22.32) | 5123 (17.69) | 0.116 |
| Time Limited Minor: Primary Infection (ADG2) | 2867 (39.60) | 10396 (35.90) | 0.076 |
| Time Limited Major (ADG3) | 979 (13.52) | 1503 (5.19) | 0.289 |
| Time Limited Major: Primary Infection (ADG4) | 1161 (16.04) | 2474 (8.54) | 0.223 |
| Allergies (ADG5) | 342 (4.62) | 1458 (5.03) | 0.014 |
| Asthma (ADG6) | 366 (5.06) | 1122 (3.87) | 0.057 |
| Likely to recur: Discrete (ADG7) | 2477 (34.21) | 7631 (26.35) | 0.172 |
| Likely to recur: Discrete infection (ADG8) | 1111 (15.35) | 3420 (11.81) | 0.103 |
| Likely to recur Progressive (ADG9) | 587 (8.11) | 812 (2.80) | 0.235 |
| Chronic Medical: Stable (ADG10) | 3261 (45.04) | 11493 (39.69) | 0.109 |
| Chronic Medical: Unstable (ADG11) | 2226 (30.75) | 5434 (18.76) | 0.28 |
| Chronic Specialty: Stable-Orthopedic (ADG12) | 241 (3.33) | 734 (2.53) | 0.047 |
| Chronic Specialty: Stable-Ear, Nose, Throat (ADG13) | Sup | Sup | Sup |
| Chronic Specialty-Stable-Eye (ADG14) | 398 (5.50) | 1409 (4.87) | 0.029 |
| Chronic Specialty: Unstable-Orthopedic (ADG16) | 277 (3.83) | 638 (2.20) | 0.095 |
| Chronic Specialty: Unstable-Ear, Nose, Throat (ADG17) | 277 (3.83) | 638 (2.20) | 0.095 |
| Chronic Specialty: Unstable-Eye (ADG18) | 1477 (5.10) | 399 (5.51) | 0.018 |
| Dermatologic (ADG20) | 778 (10.75) | 3405 (11.76) | 0.032 |
| Injuries/Adverse effects: Minor (ADG21) | 2274 (31.41) | 5359 (18.50) | 0.302 |
| Injuries/Adverse effects: Major (ADG22) | 2622 (36.22) | 4224 (14.59) | 0.513 |
| Psychosocial: Time Limited, Minor (ADG23) | 946 (13.07) | 959 (3.31) | 0.362 |
| Psychosocial: Recurrent or Persistent, Stable (ADG24) | 4289 (59.24) | 5547 (19.15) | 0.901 |
| Psychosocial: Recurrent or Persistent, Unstable (ADG25) | 2512 (34.70) | 1596 (5.51) | 0.782 |
| Signs/Symptoms: Minor (ADG26) | 2965 (40.95) | 8272 (28.56) | 0.262 |
| Signs/Symptoms: Uncertain (ADG27) | 4067 (56.17) | 11819 (40.81) | 0.311 |
| Signs/Symptoms: Major (ADG28) | 2334 (32.24) | 5704 (19.70) | 0.289 |
| Discretionary (ADG29) | 1311 (18.11) | 4424 (15.28) | 0.076 |
| See and Reassure (ADG30) | 173 (2.39) | 571 (1.97) | 0.039 |
| Prevention/Administrative (ADG31) | 2124 (29.34) | 8425 (29.09) | 0.005 |
| Malignancy (ADG32) | 700 (9.67) | 1955 (6.75) | 0.106 |
| Dental (ADG34) | 278 (3.84) | 481 (63.37) | 0.134 |
| Prior suicide attempt | 1076 (88.05) | 146 (11.95) | 0.560 |

**Supplementary Table 6: Descriptive Statistics (Female only)**

| Variables | Cases  (N=2,608) | Controls (N=10,432) | Standardized Difference |
| --- | --- | --- | --- |
| Age | 48.36 (16.05) | 48.36 (16.04) | 0 |
| Migration status |  |  |  |
| Non-immigrant | 2357 (90.38) | 8680 (83.21) | 0.213 |
| Immigrant | 201 (7.71) | 1493 (14.31) | 0.212 |
| Refugee | 50 (1.92) | 259 (2.48) | 0.039 |
| Neighbourhood Income Quintile |  |  |  |
| Q1 (lowest) | 754 (29.13) | 1915 (18.44) | 0.250 |
| Q2 | 541 (20.90) | 2015 (19.41) | 0.036 |
| Q3 | 459 (17.74) | 2115 (20.37) | 0.068 |
| Q4 | 436 (16.46) | 2166 (20.86) | 0.114 |
| Q5 (highest) | 408 (15.77) | 2172 (20.92) | 0.134 |
| Neighbourhood Instability Index Quintile |  |  |  |
| 1 (lowest instability) | 302 (11.83) | 2203 (21.29) | 0.260 |
| 2 | 381 (14.93) | 1969 (19.03) | 0.114 |
| 3 | 416 (16.30) | 1944 (18.79) | 0.071 |
| 4 | 505 (19.79) | 1989 (19.22) | 0.008 |
| 5 (highest instability) | 948(37.15) | 2243 (21.68) | 0.332 |
| Dependency Quintile |  |  |  |
| 1 (lowest dependency) | 622 (24.37) | 2480 (23.97) | 0.002 |
| 2 | 471 (18.46) | 2099 (20.28) | 0.053 |
| 3 | 449 (17.59) | 1894 (18.30) | 0.025 |
| 4 | 427 (16.73) | 1812 (17.51) | 0.027 |
| 5 (highest dependency) | 583 (22.84) | 2063 (19.94) | 0.063 |
| Time Limited Minor (ADG1) | 912 (34.97) | 2917 (27.96) | 0.151 |
| Time Limited Minor: Primary Infection (ADG2) | 1375 (52.72) | 4898 (46.95) | 0.116 |
| Time Limited Major (ADG3) | 497 (19.06) | 560 (5.37) | 0.428 |
| Time Limited Major: Primary Infection (ADG4) | 536 (20.55) | 872 (8.36) | 0.352 |
| Allergies (ADG5) | 201 (7.71) | 784 (7.52) | 0.007 |
| Asthma (ADG6) | 254 (9.74) | 615 (5.90) | 0.144 |
| Likely to recur: Discrete (ADG7) | 1331 (51.04) | 3730 (35.76) | 0.312 |
| Likely to recur: Discrete infection (ADG8) | 780 (29.91) | 2289 (21.94) | 0.183 |
| Likely to recur Progressive (ADG9) | 265 (10.16) | 205 (1.97) | 0.349 |
| Chronic Medical: Stable (ADG10) | 1374 (52.68) | 4372 (41.91) | 0.217 |
| Chronic Medical: Unstable (ADG11) | 968 (37.12) | 1820 (17.45) | 0.453 |
| Chronic Specialty: Stable-Orthopedic (ADG12) | 97 (3.72) | 257 (2.46) | 0.073 |
| Chronic Specialty: Stable-Ear, Nose, Throat (ADG13) | 64 (2.45) | 202 (1.94) | 0.035 |
| Chronic Specialty-Stable-Eye (ADG14) | 162 (6.21) | 549 (5.26) | 0.041 |
| Chronic Specialty: Unstable-Orthopedic (ADG16) | 141 (5.41) | 236 (2.26) | 0.164 |
| Chronic Specialty: Unstable-Ear, Nose, Throat (ADG17) | SUP | SUP | SUP |
| Chronic Specialty: Unstable-Eye (ADG18) | 180 (6.90) | 586 (5.62) | 0.053 |
| Dermatologic (ADG20) | 392 (15.03) | 1576 (15.11) | 0.002 |
| Injuries/Adverse effects: Minor (ADG21) | 1003 (38.46) | 2072 (19.86) | 0.418 |
| Injuries/Adverse effects: Major (ADG22) | 1282 (49.16) | 1441 (13.81) | 0.823 |
| Psychosocial: Time Limited, Minor (ADG23) | 530 (20.32) | 472 (4.52) | 0.493 |
| Psychosocial: Recurrent or Persistent, Stable (ADG24) | 2049 (78.57) | 2893 (27.73) | 1.18 |
| Psychosocial: Recurrent or Persistent, Unstable (ADG25) | 1243 (47.66) | 498 (4.77) | 1.12 |
| Signs/Symptoms: Minor (ADG26) | 1499 (57.48) | 3799 (36.42) | 0.432 |
| Signs/Symptoms: Uncertain (ADG27) | 1865 (71.51) | 5472 (52.45) | 0.40 |
| Signs/Symptoms: Major (ADG28) | 1416 (54.29) | 3752 (35.97) | 0.375 |
| Discretionary (ADG29) | 619 (23.73) | 1858 (17.81) | 0.146 |
| See and Reassure (ADG30) | 70 (2.68) | 231 (2.21) | 0.030 |
| Prevention/Administrative (ADG31) | 1203 (46.13) | 4725 (45.29) | 0.017 |
| Malignancy (ADG32) | 228 (8.74) | 718 (6.88) | 0.069 |
| Pregnancy (ADG33) | 123 (4.72) | 625 (6.02) | 0.029 |
| Dental (ADG34) | 108 (4.14) | 173 (1.66) | 0.148 |
| Prior suicide attempt | 756 (91.30) | 1852 (15.17) | 0.868 |

**Supplementary Table 7: Unadjusted logistic regression predicting suicide**

|  | Hours OR (95% CI) |
| --- | --- |
| *General Hospital* |  |
| Males: Death by suicide | 1.11 (0.90, 1.37) |
| Females: Death by suicide | 0.23 (0.15, 0.36) |
| *Psychiatric Hospital* |  |
| Males: Death by suicide | 1.04 (1.03, 1.05) |
| Females: Death by suicide | 1.00 (0.98, 1.02) |

**Supplementary Table 8: Adjusted relationship between travel time to care and suicide among males, stratified by rural and urban populations**

|  | Hours OR (95% CI) |
| --- | --- |
| *General Hospital* |  |
| Rural | 1.38 (0.65, 2.93) |
| Urban | 1.88 (1.25, 2.84) |
| *Psychiatric Hospital* |  |
| Rural | 1.00 (0.97, 1.03) |
| Urban | 1.05 (1.04, 1.06) |
